# 1-(2-Oxo-3,4-dihydro-2*H*-1,3-benzoxazin-4-yl)urea monohydrate

**DOI:** 10.1107/S1600536810032411

**Published:** 2010-08-18

**Authors:** Malahat M. Kurbanova, Aysel B. Novruzova, Atash V. Gurbanov, Sheyda R. Ismaylova, Abel M. Maharramov

**Affiliations:** a Baku State University, Z. Khalilov Street 23, Baku, AZ-1148, Azerbaijan

## Abstract

The organic molecule in the title hydrate, C_9_H_9_N_3_O_3_·H_2_O, was obtained by the condenstation of salicylic aldehyde with urea in acetonitrile. The oxazine ring adopts a slightly distorted sofa conformation, with the N atom deviating from the plane passing through the other atoms of the ring by 0.267 (2) Å. The crystal structure displays inter­molecular N—H⋯O and O—H⋯O hydrogen bonding.

## Related literature

For details of the condensation of salicyl aldehyde with urea, see: Pandey *et al.* (2008 ▶); El-Hamouly *et al.* (2007 ▶); Bobowski & Shavel (1967 ▶).
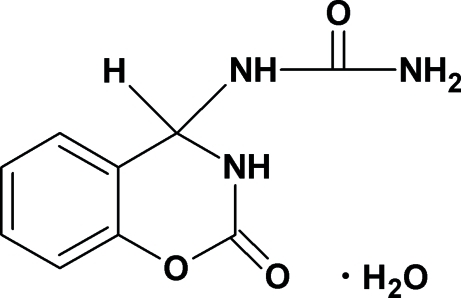


## Experimental

### 

#### Crystal data

C_9_H_9_N_3_O_3_·H_2_O
*M* *_r_* = 225.21
Monoclinic, 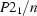

*a* = 5.3393 (10) Å
*b* = 8.5465 (16) Å
*c* = 21.846 (4) Å
β = 95.472 (4)°
*V* = 992.3 (3) Å^3^
*Z* = 4
Mo *K*α radiation
μ = 0.12 mm^−1^
*T* = 296 K
0.30 × 0.30 × 0.30 mm

#### Data collection

Bruker APEXII CCD diffractometer
Absorption correction: multi-scan (*SADABS*; Sheldrick, 2003 ▶) *T*_min_ = 0.965, *T*_max_ = 0.965
10468 measured reflections
2448 independent reflections
1942 reflections with *I* > 2σ(*I*)
*R*_int_ = 0.058

#### Refinement

*R*[*F* ^2^ > 2σ(*F* ^2^)] = 0.043
*wR*(*F* ^2^) = 0.117
*S* = 1.00
2448 reflections
145 parameters
H-atom parameters constrained
Δρ_max_ = 0.28 e Å^−3^
Δρ_min_ = −0.29 e Å^−3^

### 

Data collection: *APEX2* (Bruker, 2005 ▶); cell refinement: *SAINT-Plus* (Bruker, 2001 ▶); data reduction: *SAINT-Plus*; program(s) used to solve structure: *SHELXTL* (Sheldrick, 2008 ▶); program(s) used to refine structure: *SHELXTL*; molecular graphics: *SHELXTL*; software used to prepare material for publication: *SHELXTL*.

## Supplementary Material

Crystal structure: contains datablocks global, I. DOI: 10.1107/S1600536810032411/rk2226sup1.cif

Structure factors: contains datablocks I. DOI: 10.1107/S1600536810032411/rk2226Isup2.hkl

Additional supplementary materials:  crystallographic information; 3D view; checkCIF report

## Figures and Tables

**Table 1:** Hydrogen-bond geometry (Å, °)

*D*—H⋯*A*	*D*—H	H⋯*A*	*D*⋯*A*	*D*—H⋯*A*
N1—H1⋯O2^i^	0.90	2.05	2.946 (2)	174
N2—H2*A*⋯O3^ii^	0.90	2.41	3.088 (2)	132
N3—H3*A*⋯O4^iii^	0.93	2.14	3.054 (2)	169
N3—H3*B*⋯O4	0.96	1.99	2.923 (2)	166
O4—H4*A*⋯O2^iv^	0.94	1.91	2.774 (2)	153
O4—H4*B*⋯O3^v^	0.94	1.94	2.842 (2)	161

Symmetry codes: (i) 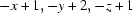; (ii) 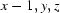; (iii) 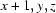; (iv) 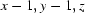; (v) 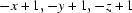.

**Fig. 1.**
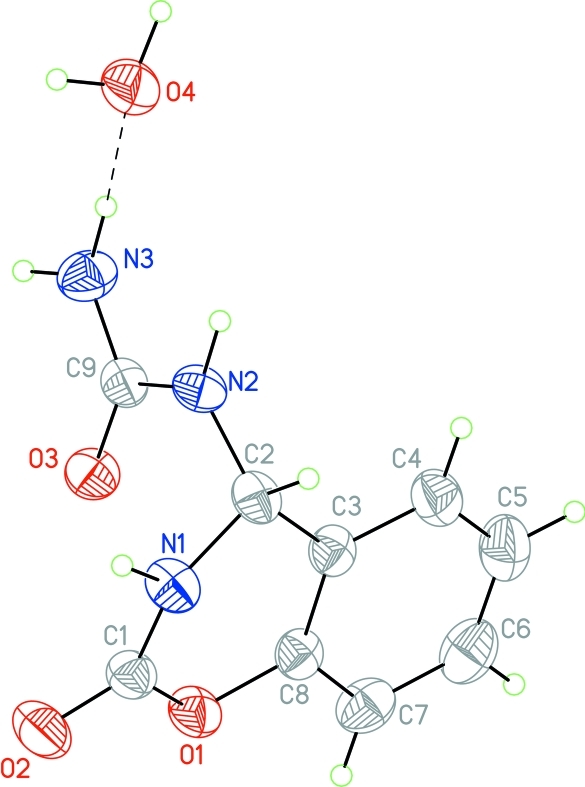
Molecular structure of I with the atom numbering scheme. Displacement ellipsoids are drawn at the 50% proabbility level. H atoms are presented as a small spheres of arbitrary radius.

**Fig. 2.**
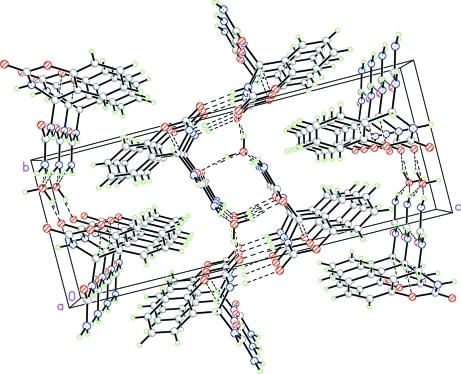
Crystal packing of the molecules of I viewed down the a axis. Dashed lines show intermolecular hydrogen bonds.

**Fig. 3.**
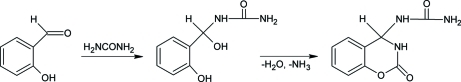
Condensation of salicyl aldehyde with two molecules of urea with reception benzoxazin.

## supplementary crystallographic information

### Comment

3,4-Dihydro-3-alkyl-2*H*-1,3-benzoxazin-2-ones, 4,4'-oxobis(3,4-dihydro-3-alkyl-2*H*-benzoxazin-2-ones), and 1-(3,4-dihydro-3-alkyl-2-oxo-2*H*-1,3-benzoxazin-4-yl-1,3-dialkylureas were obtained by the condensation of *o*-hydroxyaromatic aldehydes with alkylisocyanates (Bobowski & Shavel, 1967). The formation 1,1'-[(2-hydroxyphenyl)methylene]diurea by the condensation of salicyl aldehyde with two molecules of carbamide is well known from the literature (El-Hamouly *et al.*, 2007; Pandey *et al.*, 2008).

The title molecule contains a fused bicycle system containing two six-membered rings (benzene and oxazin) (Fig. 1). The oxazin cycle adopts a slightly distorted *sofa* conformation, with the nitrogen N1 atom deviating from plane passed through the other atoms of the cycle by 0.267 (2)Å. Such disposion of the nitrogen atom is defined by the intermolecular N1–H1···O2^i^ hydrogen bonding (Table 1). Symmetry code: (i) -*x*+1, -*y*+2, -*z*+1. The nitrogen N1 atom has a pyramidalized configuration (sum of bond angles at the nitrogen N1 atom is 352.7°, the C2–N1–O1–C1 torsion angle is 22.4 (2)°). The carbamide substituent of the oxazin cycle is in axial position (the C9–N2–C2–N1 torsion angle is -64.31 (15)°).

Compound I possesses one asymmetric center at the C2, but the crystal of I is racemate.

In crystal of I, the organic molecules and solvate water molecules are bound into complex two-tier layers parallel to (0 0 1) by the hydrogen bonds (Fig. 2, Table 1).

### Experimental

The solution of salicylic aldehyde (0.02 mol), urea (0.03 mol), 1 ml trichloroacetic acid, 3 ml *Ac*OH in 15 ml acetonitril was mixed for 3-4 h (Fig. 3). Of a reaction course observed using thin layer chromatography (*TLC*) method. After cooling the product was filtered and washed out by ethanol. The target product was obtained by re-crystallization of a water solution of ethanol (1:3) as white crystals. *M*.p. = 498-500 K. IR, ν/cm^-1^: 1456-1541 (C═C), 1646 (NH–*C(O*)–NH_2_), 1710 (*C(O)*–O), 3349 (NH(lactam), associated), 3346 (C(O)–*NH_2_*). Mass spectrum, m/*z*: 230 [*M*+Na]^+. 1^H NMR ((CD_3_)_2_SO, 293 K): 8.5 (S, 1H, NH), 7.3(m, 2H, H6, H7), 7.15 (m, 2H, H3, H5),7.05 (d, 1H, H8, ^3^J = 7.2), 6.2 (d, 1H, H4, ^3^J = 7.5), 5.65 (S, 2H, NH_2_). ^13^C NMR ((CD_3_)_2_SO, 293 K): δ = 164 (S, NH–C(O)–NH_2_), 158 (S, C(O)–O), 150 (S, C9), 130 (S, C8), 127 (S, C5), 124.5 (S, C7), 121 (S, C10), 116 (S, C6), 58 (S, C4).

### Refinement

The amino-H atoms as well as H-atoms of the water solvate molecule were localized in the difference Fourier map and included in the refinement with fixed positional and isotropic displacement parameters [*U*_iso_(H) = 1.2*U*_eq_(N) and *U*_iso_(H) = 1.5*U*_eq_(O)]. The other hydrogen atoms were placed in calculated positions with C–H = 0.93-0.98Å and refined in the riding model with fixed isotropic displacement parameters [*U*_iso_(H) = 1.2*U*_eq_(C)].

### Crystal data

**Table:**

C_9_H_9_N_3_O_3_·H_2_O	*F*(000) = 472
*M**_r_* = 225.21	*D*_x_ = 1.508 Mg m^−^^3^
Monoclinic, *P*2_1_/*n*	Melting point: 499 K
Hall symbol: -P 2yn	Mo *K*α radiation, λ = 0.71073 Å
*a* = 5.3393 (10) Å	Cell parameters from 3341 reflections
*b* = 8.5465 (16) Å	θ = 2.6–28.2°
*c* = 21.846 (4) Å	µ = 0.12 mm^−^^1^
β = 95.472 (4)°	*T* = 296 K
*V* = 992.3 (3) Å^3^	Prism, colourless
*Z* = 4	0.30 × 0.30 × 0.30 mm

### Data collection

**Table:**

Bruker APEXII CCD diffractometer	2448 independent reflections
Radiation source: fine-focus sealed tube	1942 reflections with *I* > 2σ(*I*)
graphite	*R*_int_ = 0.058
φ and ω scans	θ_max_ = 28.4°, θ_min_ = 1.9°
Absorption correction: multi-scan (*SADABS*; Sheldrick, 2003)	*h* = −7→7
*T*_min_ = 0.965, *T*_max_ = 0.965	*k* = −11→11
10468 measured reflections	*l* = −29→28

### Refinement

**Table:**

Refinement on *F*^2^	Primary atom site location: structure-invariant direct methods
Least-squares matrix: full	Secondary atom site location: difference Fourier map
*R*[*F*^2^ > 2σ(*F*^2^)] = 0.043	Hydrogen site location: difference Fourier map
*wR*(*F*^2^) = 0.117	H-atom parameters constrained
*S* = 1.00	*w* = 1/[σ^2^(*F*_o_^2^) + (0.0598*P*)^2^ + 0.12*P*] where *P* = (*F*_o_^2^ + 2*F*_c_^2^)/3
2448 reflections	(Δ/σ)_max_ < 0.001
145 parameters	Δρ_max_ = 0.28 e Å^−^^3^
0 restraints	Δρ_min_ = −0.29 e Å^−^^3^

### Special details

**Table:**

Geometry. All s.u.'s (except the s.u. in the dihedral angle between two l.s. planes) are estimated using the full covariance matrix. The cell s.u.'s are taken into account individually in the estimation of s.u.'s in distances, angles and torsion angles; correlations between s.u.'s in cell parameters are only used when they are defined by crystal symmetry. An approximate (isotropic) treatment of cell s.u.'s is used for estimating s.u.'s involving l.s. planes.
Refinement. Refinement of *F*^2^ against ALL reflections. The weighted *R*-factor *wR* and goodness of fit *S* are based on *F*^2^, conventional *R*-factors *R* are based on *F*, with *F* set to zero for negative *F*^2^. The threshold expression of *F*^2^ > σ(*F*^2^) is used only for calculating *R*-factors(gt) *etc*. and is not relevant to the choice of reflections for refinement. *R*-factors based on *F*^2^ are statistically about twice as large as those based on *F*, and *R*-factors based on ALL data will be even larger.

### Fractional atomic coordinates and isotropic or equivalent isotropic displacement parameters (Å^2^)

**Table:**

	*x*	*y*	*z*	*U*_iso_*/*U*_eq_	
O1	0.72114 (16)	0.97940 (12)	0.35888 (4)	0.0372 (2)	
O2	0.7419 (2)	1.05038 (14)	0.45587 (5)	0.0500 (3)	
O3	0.74592 (16)	0.64789 (12)	0.40776 (4)	0.0377 (3)	
N1	0.3995 (2)	0.91465 (14)	0.41776 (5)	0.0360 (3)	
H1	0.3548	0.9179	0.4565	0.043*	
N2	0.32112 (19)	0.63842 (14)	0.40155 (5)	0.0348 (3)	
H2A	0.1839	0.5802	0.4065	0.042*	
N3	0.5568 (2)	0.42106 (15)	0.43002 (6)	0.0420 (3)	
H3A	0.7098	0.3794	0.4466	0.050*	
H3B	0.4077	0.3652	0.4378	0.050*	
C1	0.6227 (2)	0.98097 (16)	0.41376 (6)	0.0337 (3)	
C2	0.2868 (2)	0.79512 (16)	0.37637 (6)	0.0322 (3)	
H2	0.1054	0.8154	0.3709	0.039*	
C3	0.3850 (2)	0.81370 (15)	0.31454 (6)	0.0316 (3)	
C4	0.2696 (3)	0.74090 (18)	0.26224 (7)	0.0418 (3)	
H4	0.1274	0.6796	0.2652	0.050*	
C5	0.3641 (3)	0.7586 (2)	0.20596 (7)	0.0495 (4)	
H5	0.2852	0.7098	0.1713	0.059*	
C6	0.5756 (3)	0.8489 (2)	0.20131 (7)	0.0483 (4)	
H6	0.6398	0.8600	0.1634	0.058*	
C7	0.6926 (3)	0.92281 (19)	0.25240 (7)	0.0418 (3)	
H7	0.8347	0.9842	0.2493	0.050*	
C8	0.5943 (2)	0.90387 (16)	0.30837 (6)	0.0316 (3)	
C9	0.5510 (2)	0.57119 (16)	0.41307 (5)	0.0306 (3)	
O4	0.08489 (19)	0.29496 (14)	0.46692 (5)	0.0499 (3)	
H4A	0.0070	0.1995	0.4555	0.075*	
H4B	0.1116	0.2988	0.5099	0.075*	

### Atomic displacement parameters (Å^2^)

**Table:**

	*U*^11^	*U*^22^	*U*^33^	*U*^12^	*U*^13^	*U*^23^
O1	0.0276 (4)	0.0451 (6)	0.0392 (5)	−0.0083 (4)	0.0049 (4)	0.0007 (4)
O2	0.0477 (6)	0.0588 (7)	0.0432 (6)	−0.0203 (5)	0.0027 (5)	−0.0081 (5)
O3	0.0229 (4)	0.0452 (6)	0.0455 (5)	−0.0048 (4)	0.0059 (4)	0.0060 (4)
N1	0.0293 (5)	0.0432 (7)	0.0368 (6)	−0.0030 (5)	0.0093 (4)	−0.0067 (5)
N2	0.0218 (5)	0.0415 (6)	0.0419 (6)	−0.0091 (4)	0.0066 (4)	0.0025 (5)
N3	0.0385 (6)	0.0410 (7)	0.0477 (7)	−0.0032 (5)	0.0101 (5)	0.0076 (5)
C1	0.0295 (6)	0.0344 (7)	0.0371 (7)	−0.0026 (5)	0.0024 (5)	0.0001 (5)
C2	0.0177 (5)	0.0416 (7)	0.0375 (7)	−0.0009 (5)	0.0041 (5)	−0.0021 (5)
C3	0.0240 (5)	0.0363 (7)	0.0343 (6)	0.0033 (5)	0.0019 (5)	0.0020 (5)
C4	0.0352 (7)	0.0466 (8)	0.0423 (8)	−0.0025 (6)	−0.0023 (6)	−0.0008 (6)
C5	0.0556 (9)	0.0565 (10)	0.0349 (7)	0.0027 (7)	−0.0032 (6)	−0.0035 (7)
C6	0.0578 (9)	0.0539 (9)	0.0346 (7)	0.0087 (7)	0.0121 (6)	0.0041 (7)
C7	0.0385 (7)	0.0448 (8)	0.0437 (8)	0.0023 (6)	0.0125 (6)	0.0086 (6)
C8	0.0248 (5)	0.0350 (7)	0.0350 (6)	0.0040 (5)	0.0025 (5)	0.0023 (5)
C9	0.0269 (6)	0.0396 (7)	0.0260 (6)	−0.0056 (5)	0.0062 (4)	−0.0013 (5)
O4	0.0416 (6)	0.0568 (7)	0.0506 (6)	−0.0155 (5)	0.0009 (5)	0.0044 (5)

### Geometric parameters (Å, °)

**Table:**

O1—C1	1.3541 (16)	C2—H2	0.9800
O1—C8	1.3965 (16)	C3—C8	1.3746 (18)
O2—C1	1.2213 (17)	C3—C4	1.3919 (19)
O3—C9	1.2449 (15)	C4—C5	1.381 (2)
N1—C1	1.3300 (16)	C4—H4	0.9300
N1—C2	1.4558 (17)	C5—C6	1.380 (2)
N1—H1	0.9026	C5—H5	0.9300
N2—C9	1.3567 (17)	C6—C7	1.379 (2)
N2—C2	1.4527 (18)	C6—H6	0.9300
N2—H2A	0.9010	C7—C8	1.3853 (19)
N3—C9	1.3350 (19)	C7—H7	0.9300
N3—H3A	0.9321	O4—H4A	0.9390
N3—H3B	0.9572	O4—H4B	0.9361
C2—C3	1.5032 (18)		
			
C1—O1—C8	120.28 (10)	C4—C3—C2	121.70 (12)
C1—N1—C2	125.32 (11)	C5—C4—C3	120.75 (14)
C1—N1—H1	111.6	C5—C4—H4	119.6
C2—N1—H1	118.3	C3—C4—H4	119.6
C9—N2—C2	122.67 (10)	C6—C5—C4	119.86 (14)
C9—N2—H2A	118.4	C6—C5—H5	120.1
C2—N2—H2A	118.7	C4—C5—H5	120.1
C9—N3—H3A	118.1	C7—C6—C5	120.49 (14)
C9—N3—H3B	122.1	C7—C6—H6	119.8
H3A—N3—H3B	117.0	C5—C6—H6	119.8
O2—C1—N1	124.29 (12)	C6—C7—C8	118.70 (14)
O2—C1—O1	116.96 (12)	C6—C7—H7	120.7
N1—C1—O1	118.66 (11)	C8—C7—H7	120.6
N2—C2—N1	112.46 (11)	C3—C8—C7	122.14 (13)
N2—C2—C3	113.30 (11)	C3—C8—O1	121.28 (11)
N1—C2—C3	108.97 (10)	C7—C8—O1	116.57 (12)
N2—C2—H2	107.3	O3—C9—N3	122.31 (12)
N1—C2—H2	107.3	O3—C9—N2	120.61 (12)
C3—C2—H2	107.3	N3—C9—N2	117.07 (11)
C8—C3—C4	118.06 (12)	H4A—O4—H4B	108.6
C8—C3—C2	120.24 (11)		
			
C2—N1—C1—O2	−161.10 (14)	C3—C4—C5—C6	0.2 (2)
C2—N1—C1—O1	22.4 (2)	C4—C5—C6—C7	−0.5 (3)
C8—O1—C1—O2	−179.29 (12)	C5—C6—C7—C8	0.3 (2)
C8—O1—C1—N1	−2.56 (18)	C4—C3—C8—C7	−0.5 (2)
C9—N2—C2—N1	−64.31 (15)	C2—C3—C8—C7	179.31 (12)
C9—N2—C2—C3	59.82 (15)	C4—C3—C8—O1	−179.47 (12)
C1—N1—C2—N2	98.81 (15)	C2—C3—C8—O1	0.31 (19)
C1—N1—C2—C3	−27.67 (17)	C6—C7—C8—C3	0.2 (2)
N2—C2—C3—C8	−110.70 (13)	C6—C7—C8—O1	179.23 (13)
N1—C2—C3—C8	15.31 (16)	C1—O1—C8—C3	−8.08 (18)
N2—C2—C3—C4	69.07 (15)	C1—O1—C8—C7	172.86 (12)
N1—C2—C3—C4	−164.92 (12)	C2—N2—C9—O3	6.81 (19)
C8—C3—C4—C5	0.3 (2)	C2—N2—C9—N3	−173.09 (11)
C2—C3—C4—C5	−179.52 (14)		

### Hydrogen-bond geometry (Å, °)

**Table:**

*D*—H···*A*	*D*—H	H···*A*	*D*···*A*	*D*—H···*A*
N1—H1···O2^i^	0.90	2.05	2.946 (2)	174
N2—H2A···O3^ii^	0.90	2.41	3.088 (2)	132
N3—H3A···O4^iii^	0.93	2.14	3.054 (2)	169
N3—H3B···O4	0.96	1.99	2.923 (2)	166
O4—H4A···O2^iv^	0.94	1.91	2.774 (2)	153
O4—H4B···O3^v^	0.94	1.94	2.842 (2)	161

Symmetry codes: (i) −*x*+1, −*y*+2, −*z*+1; (ii) *x*−1, *y*, *z*; (iii) *x*+1, *y*, *z*; (iv) *x*−1, *y*−1, *z*; (v) −*x*+1, −*y*+1, −*z*+1.
